# Nimotuzumab Site-Specifically Labeled with ^89^Zr and ^225^Ac Using SpyTag/SpyCatcher for PET Imaging and Alpha Particle Radioimmunotherapy of Epidermal Growth Factor Receptor Positive Cancers

**DOI:** 10.3390/cancers12113449

**Published:** 2020-11-20

**Authors:** Viswas Raja Solomon, Kris Barreto, Wendy Bernhard, Elahe Alizadeh, Patrick Causey, Randy Perron, Denise Gendron, Md. Kausar Alam, Adriana Carr, C. Ronald Geyer, Humphrey Fonge

**Affiliations:** 1 Department of Medical Imaging, College of Medicine, University of Saskatchewan, Saskatoon, SK S7N 0W8, Canada; vrajasolomon@gmail.com (V.R.S.); elahe.alizadeh@queensu.ca (E.A.); 2 Department of Pathology and Laboratory Medicine, College of Medicine, University of Saskatchewan, Saskatoon, SK S7N 5E5, Canada; dr.barreto@gmail.com (K.B.); wendylynnette@gmail.com (W.B.); alambt01@yahoo.com (M.K.A.); 3 Canadian Nuclear Laboratories, Chalk River, ON K0J 1J0, Canada; patrick.causey@cnl.ca (P.C.); randy.perron@cnl.ca (R.P.); denise.gendron@cnl.ca (D.G.); 4 Research and Development Direction, Center of Molecular Immunology, 216 Street and 15 Avenue, Atabey, Playa, P.O. Box 16040, Havana 11600, Cuba; adriana@cim.sld.cu; 5 Department of Medical Imaging, Royal University Hospital, Saskatoon, SK S7N 0W8, Canada

**Keywords:** site-specific labeling, SpyTag/∆N-SpyCatcher, radioimmunotherapy, diagnostic, immunoPET, EGFR

## Abstract

**Simple Summary:**

Monoclonal antibodies (IgG) are excellent probes for targeting cell surface receptors for imaging and therapeutic applications. These theranostic agents are often developed by randomly conjugating radioisotopes/drugs/chelators to the primary amine of lysine or the sulfhydryl groups of cysteine on the antibody. Random conjugation often alters the properties of the antibody. We have site-specifically radiolabeled nimotuzumab an anti-epidermal growth factor receptor (EGFR) monoclonal antibody with 89Zr and 225Ac using SpyTag: ∆N-SpyCatcher for positron emission tomography (PET) imaging and alpha particle radiotherapy, and evaluated these agents in a model of EGFR-positive triple negative breast cancer (TNBC). Nimotuzumab-SpyTag-∆N-SpyCatcher constructs showed improved binding in vitro compared with randomly conjugated constructs. 89Zr-nimotuzumab-SpyTag-∆N-SpyCatcher specifically delineated EGFR-positive xenograft in vivo using microPET/CT imaging. Compared with control treatment groups, 225Ac-nimotuzumab-SpyTag-∆N-SpyCatcher more than doubled the survival of mice bearing EGFR-positive MDA-MB-231 TNBC xenograft. This work highlights a facile method to site-specifically radiolabel antibodies using SpyTag: ∆N-SpyCatcher.

**Abstract:**

To develop imaging and therapeutic agents, antibodies are often conjugated randomly to a chelator/radioisotope or drug using a primary amine (NH_2_) of lysine or sulfhydryl (SH) of cysteine. Random conjugation to NH_2_ or SH groups can require extreme conditions and may affect target recognition/binding and must therefore be tested. In the present study, nimotuzumab was site-specifically labeled using ∆N-SpyCatcher/SpyTag with different chelators and radiometals. Nimotuzumab is a well-tolerated anti-EGFR antibody with low skin toxicities. First, ΔN-SpyCatcher was reduced using tris(2-carboxyethyl)phosphine (TCEP), which was followed by desferoxamine-maleimide (DFO-mal) conjugation to yield a reactive ΔN-SpyCatcher-DFO. The ΔN-SpyCatcher-DFO was reacted with nimotuzumab-SpyTag to obtain stable nimotuzumab-SpyTag-∆N-SpyCatcher-DFO. Radiolabeling was performed with ^89^Zr, and the conjugate was used for the in vivo microPET imaging of EGFR-positive MDA-MB-468 xenografts. Similarly, ∆N-SpyCatcher was conjugated to an eighteen-membered macrocyclic chelator macropa-maleimide and used to radiolabel nimotuzumab-SpyTag with actinium-225 (^225^Ac) for in vivo radiotherapy studies. All constructs were characterized using biolayer interferometry, flow cytometry, radioligand binding assays, HPLC, and bioanalyzer. MicroPET/CT imaging showed a good tumor uptake of ^89^Zr-nimotuzumab-SpyTag-∆N-SpyCatcher with 6.0 ± 0.6%IA/cc (*n* = 3) at 48 h post injection. The EC_50_ of ^225^Ac-nimotuzumab-SpyTag-∆N-SpyCatcher and ^225^Ac-control-IgG-SpyTag-∆N-SpyCatcher against an EGFR-positive cell-line (MDA-MB-468) was 3.7 ± 3.3 Bq/mL (0.04 ± 0.03 nM) and 18.5 ± 4.4 Bq/mL (0.2 ± 0.04 nM), respectively. In mice bearing MDA-MB-468 EGFR-positive xenografts, ^225^Ac-nimotuzumab-SpyTag-∆N-SpyCatcher significantly (*p* = 0.0017) prolonged the survival of mice (64 days) compared to ^225^Ac-control IgG (28.5 days), nimotuzumab (28.5 days), or PBS-treated mice (30 days). The results showed that the conjugation and labeling using SpyTag/∆N-SpyCatcher to nimotuzumab did not significantly (*p* > 0.05) alter the receptor binding of nimotuzumab compared with a non-specific conjugation approach. ^225^Ac-nimotuzumab-SpyTag-∆N-SpyCatcher was effective in vitro and in an EGFR-positive triple negative breast cancer xenograft model.

## 1. Introduction

Epidermal growth factor receptor I (EGFR) is overexpressed in all aggressive cancers of epithelial origin, including squamous cell head and neck (90–100%), glioma (90–100%), non-small cell lung (75–90%), colorectal (80–85%), breast (20–30%), and cervical cancers [1,2,3,4,5]. Anti-EGFR antibodies—e.g., cetuximab, panitumumab, necitumumab, and nimotuzumab—have been used for treating EGFR-positive cancers [6,7,8]. Except for nimotuzumab, these antibodies have all been associated with significant cutaneous toxicity in 45–100% of patients [9,10,11,12]. In contrast, nimotuzumab is better tolerated [9,13] and has low skin toxicities because of its “affinity optimized” binding characteristic, which ensures a low transient binding to low-EGFR-expressing healthy tissues such as the skin [14].

The sensitive and quantitative properties of positron emission tomography (PET) combined with the high affinity and specificity of antibodies make them the probe of choice for imaging and radiotherapeutic applications. Radiolabeling antibodies with radiometals is often carried out by conjugating the chelator randomly to one of the lysine residues on the antibody. We and many other authors have shown that random conjugation results in a decrease in the immunoreactivity and binding and may significantly change the biodistribution of the antibody [15]. Site-specific conjugation often preserves the properties of the antibody/probe and hence results in optimal in vivo characteristics [16], and is particularly important when high labeling ratios are desired [17]. Here, we report the use of the SpyTag/SpyCatcher system to site-specifically label monoclonal antibody nimotuzumab for PET imaging (using ^89^Zr) and alpha particle therapy (using ^225^Ac). We chose the SpyTag/Catcher system as it is efficient, stable, and requires minimal antibody modification. It can also be used in a variety of different buffers, pHs, etc. Applications of the SpyTag/SpyCatcher system have been previously reviewed [16]. PET imaging using ^89^Zr-labeled antibodies allows for patient stratification based on the expression of the molecular target. For example, ^89^Zr-atezolizumab PET/CT imaging was recently used to stratify patients who will respond to anti-PD-L1 therapy [18]. Therefore, there is great interest in ^89^Zr-labeled immunoPET probes with 76 ongoing clinical trials [19,20,21].

**Scheme 1:**
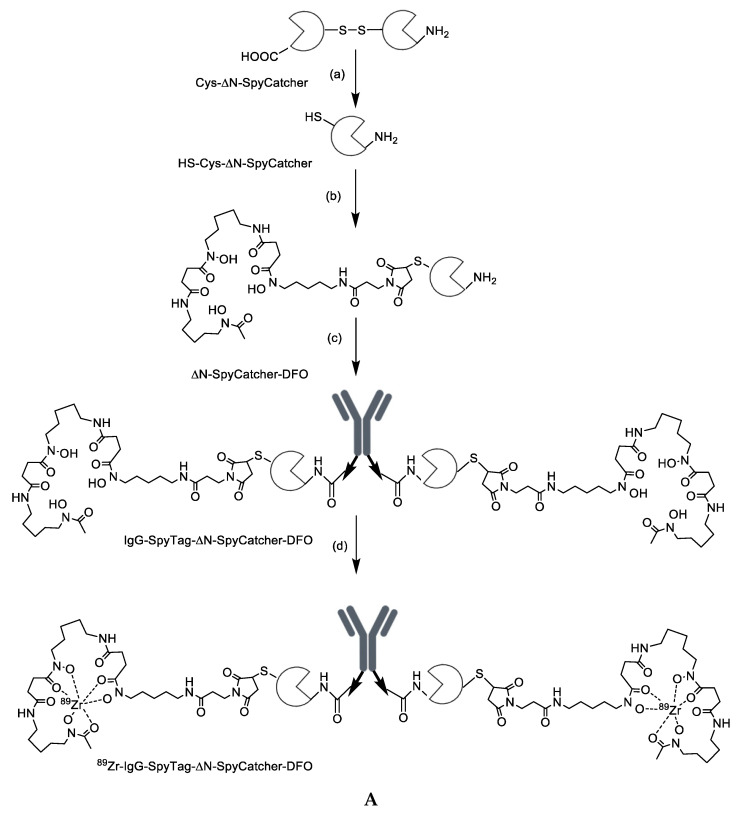

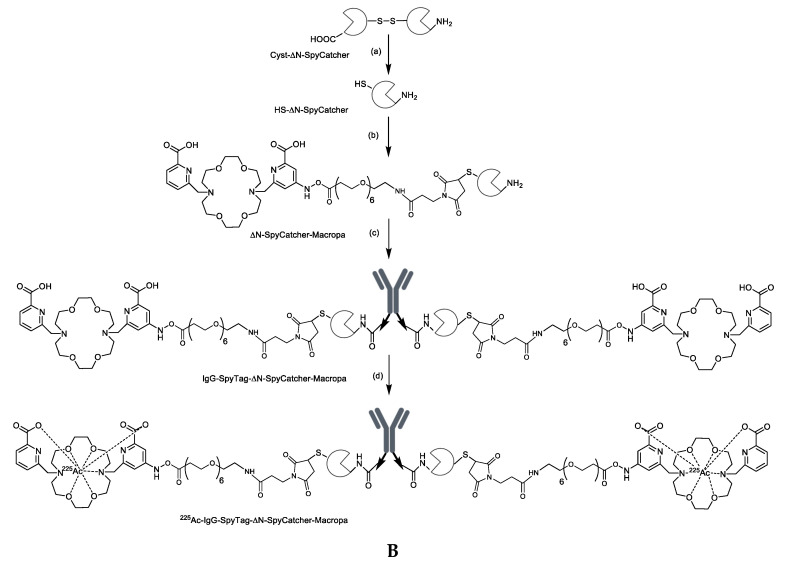
(**A**). Site-specific radiolabeling of IgG-SpyTag-ΔN-SpyCatcher with ^89^Zr: First, Cyst-ΔN-SpyCatcher was reduced with tris(2-carboxyethyl)phosphine (TCEP) to obtain reactive SH-ΔN-SpyCatcher and conjugated with deferoxamine (DFO)-maleimide to form ΔN-SpyCatcher-DFO. DFO-labeled ΔN-SpyCatcher was conjugated with SpyTag-fused antibody for site-specific labeling. Finally, IgG-SpyTag-ΔN-SpyCatcher-DFO was labeled with ^89^Zr oxalate. Reagents and conditions: (**a**) 50-fold molar excess TCEP, room temperature (RT) 2 h; (**b**) 15-fold molar excess DFO-maleimide at 4 °C overnight; (**c**) ligation of IgG-SpyTag, 3 h, RT; (**d**) -(2-Hydroxyethyl)-1-piperazineethanesulfonic acid (HEPES) buffer, sodium bicarbonate (Na_2_CO_3_), ^89^Zr-oxalate, 37 °C, 2 h. (**B**). Site-specific radiolabeling of IgG-SpyTag-ΔN-SpyCatcher with ^225^Ac: First, Cyst-ΔN-SpyCatcher was reduced using tris(2-carboxyethyl)phosphine (TCEP) to obtain reactive HS-ΔN-SpyCatcher, followed by conjugation with macropa-polyethylene glycol (PEG_6_)-maleimide to form ΔN-SpyCatcher-macropa. Then, the macropa-derivatized ΔN-SpyCatcher was conjugated with SpyTag-fused antibody for site-specific labeling. Finally, IgG-SpyTag-ΔN-SpyCatcher-macropa was labeled with ^225^Ac nitrate. Reagents and conditions: (**a**) 50-fold molar excess TCEP, RT, 2 h; (**b**) 15-fold molar excess macropa-PEG_6_-maleimide at 4 °C overnight; (**c**) ligation of IgG-SpyTag, 3 h, RT; (**d**) ^225^Ac-nitrate, 150 mM of ammonium acetate (pH 6.0, 100 µL), 37 °C, 50 min.

^225^Ac-labeled peptide and antibody radiotherapeutics also have gained significant clinical interest, with 10 of these currently in clinical development [22]. The characteristics of ^225^Ac which include a t_1/2_ of 10.0 days, and emission of 5 αs with energy 6–8 MeV (cumulatively 28 MeV/decay) within a range of 50–80 μm with a linear energy transfer (LET) of up to 0.16 MeV/μm make it ideal for antibody/peptide targeted radiotherapy. Here, we have expressed nimotuzumab as a fusion protein with SpyTag at the C-terminus. In the first application, site-specific labeling with ^89^Zr is used for microPET/CT imaging in a mouse model of EGFR-positive tumor. In the second application, nimotuzumab-SpyTag is labeled with ^225^Ac for radiotherapy. In both cases, SpyCatcher conjugates are prepared and then conjugated to the antibody. Site-specific labeling does not alter the affinity and demonstrates the use of the SpyTag/SpyCatcher (Scheme 1A,B) as a simple method for radiolabeling antibodies with different radionuclides.

## 2. Results

### 2.1. Synthesis and Characterization of Antibody-SpyTag and DFO-Cysteine-∆N-SpyCatcher

**Figure 1:**
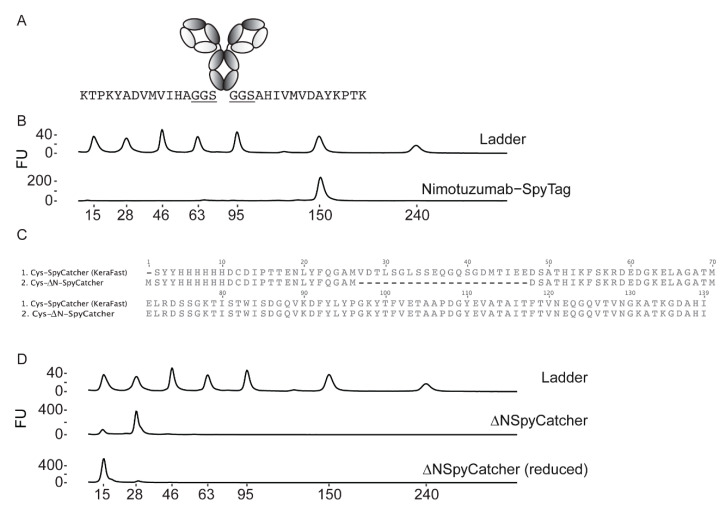
Starting biomolecules: (**A**) IgG-SpyTag. IgG is made up of two light (VL+CL) and heavy (VH+CH1+CH2+CH3) chains. The IgG is expressed with the SpyTag sequence at the C-terminus. (**B**) Bioanalyzer chromatogram of ladder and purified nimotuzumab-SpyTag (150 kDa) protein. (**C**) Sequence alignment of Cys-SpyCatcher and the Cys-ΔN-SpyCatcher construct used in this manuscript. (**D**) Bioanalyzer chromatogram of ladder and purified Cys-ΔN-SpyCatcher under non-reducing (monomer: 14.8 kDa; dimmer: 27.6 kDa) and reducing (15.2 kDa) conditions.

IgGs were expressed from human cells with the SpyTag at the C-terminal of the Fc domain, resulting in two SpyTags per IgG (Figure 1A). IgG-SpyTag fusions expressed ~2-fold worse than their non-fusion counterparts under these conditions, resulting in yields of 5 ± 1 mg/mL for a control anti-maltose binding protein IgG1 (*n* = 2) and 4 ± 1 mg/L (*n* = 5) for nimotuzumab, an anti-EGFR IgG1. The resulting IgG-SpyTags had purities of 98 ± 1% for control-IgG and 96 ± 5% for nimotuzumab, with molecular weights of 165 ± 2 kDa for control-IgG and 150 ± 1 kDa for nimotuzumab (Figure 1B).

We constructed a variant of the SpyCatcher containing a deletion in the N-terminus (∆N-SpyCatcher) (Figure 1C). To this construct, we added a single cysteine to allow chelator conjugation via maleimide chemistry (Scheme 1A,B). Conjugation chemistry involving primary amines was avoided, since a lysine from the SpyCatcher is cross-linked to an aspartic acid in the SpyTag [23] and we did not want to conjugate at this site. The Cys-∆N-SpyCatcher was expressed and purified in *E. coli*. Under non-reducing conditions, two major peaks were observed at 14.8 ± 0.1 kDa (18 ± 1%) and 27.6 ± 0.4 kDa (75 ± 5%), identified as a monomer and dimer since the reducing conditions resulted in a major 15.2 kDa (87%) peak (Figure 1D). The protein was reduced and reacted with DFO-maleimide. The reaction was purified to remove unreacted DFO and free tris(2-carboxyethyl)phosphine (TCEP). To remove the unmodified Cys-∆N-SpyCatcher, we stored the sample for 1 week at 4 °C to allow any unreacted Cys-∆N-SpyCatcher to form cysteine dimers. The sample was then purified by size exclusion chromatography (SEC) to separate the unlabeled dimer from the monomer (Figure S1), resulting in the final purified ∆N-SpyCatcher-DFO (Figure 1D).

### 2.2. Characterization of the DFO-∆N-SpyCatcher IgG-SpyTag Immunoconjugate

To avoid additional purification steps, we sought to achieve 100% labeled antibody with no free SpyTag. It has previously been shown that the SpyTag:SpyCatcher reaction is highly efficient under a variety of conditions [23]. We examined the effects of molar ratio on the percentage of heavy chain converted, since it was not known if the steric hindrance from the IgG or other factors would require an excess of ∆N-SpyCatcher-DFO to drive the reaction to completion.

**Figure 2:**
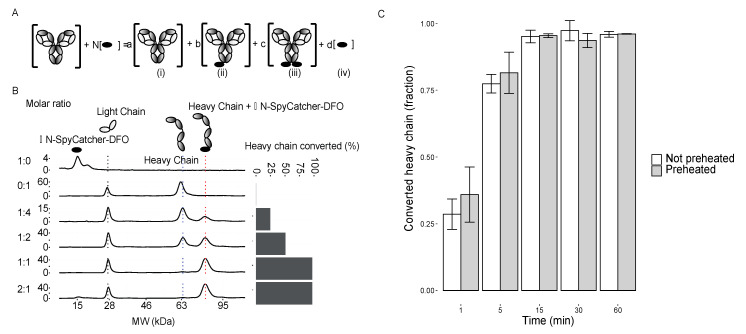
Evaluation of the bioconjugation reaction. (**A**) Overview of the reaction of IgG-SpyTag with DFO-SpyCatcher. IgG reacted with SpyCatcher can result in four products: (i) an unlabeled IgG, (ii) an IgG with one SpyCatcher, (iii) an IgG with two SpyCatchers, and (iii) unreacted SpyCatcher. (**B**) Analysis of the reaction products at different molar ratios of SpyTag: SpyCatcher. After reaction, the sample is reduced, separating the IgG into light and heavy chains. A heavy chain modified with the SpyCatcher results in an increase in MW. Representative bioanalyzer chromatograms are shown for reactions, and the quantification of peaks is shown on the right. (**C**) The kinetics of SpyCatcher-DFO with IgG-SpyTag were measured by pre-incubating the ∆N-SpyCatcher-DFO for 1 h at RT or being heated. All the reactions include IgG-SpyTag (10 µM) for the indicated times.

Nimotuzumab and a control IgG-SpyTag were tested. Each IgG has two SpyTag sites at the C-terminus that, when reacted with ∆N-SpyCatcher-DFO, could result in four possible probes: (i) unconjugated IgG, (ii) IgG conjugated with one ∆N-SpyCatcher-DFO, (iii) IgG conjugated with two ∆N-SpyCatcher-DFO, and (iv) free ∆N-SpyCatcher-DFO (Figure 2A). To determine the efficiency of the reaction and if both sites of an IgG could be labeled or if steric hindrance would interfere with labeling, we reacted the ∆N-SpyCatcher-DFO at different molar ratios with IgG-SpyTag. We analyzed the products under reducing conditions, giving a peak for the free ∆N-SpyCatcher-DFO at ~15 kDa, the light chain at ~28 kDa, the heavy chain at ~64 kDa, and the modified heavy chain at ~81 kDa (Figure 2B). When there was no ∆N-SpyCatcher-DFO present (0:1), we only saw peaks for the light chain and unreacted heavy chain, as expected. When there was a 4-fold excess of SpyTag (1:4), we saw that all ∆N-SpyCatcher-DFO was reacted, resulting in a 25% conjugation at the heavy chain. At a two-fold molar excess of SpyTag (1:2), we saw 50% of the heavy chain labeled, as expected. At equimolar concentrations (1:1), 99% of the heavy chain was converted, indicating that the reaction was efficient and there was no steric hindrance. Since the reaction plateaued at a 1:1 ratio, excess ∆N-SpyCatcher-DFO (2:1) did not result in more conversion and is not required to drive the reaction to completion (Figure 2B).

The reaction was shown to go to completion at a 1:1 molar ratio after 1 h in PBS at room temperature. A common problem with radiolabeling proteins such as IgGs is that labeling conditions such as pH and temperature can negatively affect the antibodies’ properties [24]. The SpyCatcher has already been shown to be robust to changes in the buffer and pH [25], however many radiolabeling protocols benefit from increased temperature. To determine the optimal time and how preheating the ∆N-SpyCatcher-DFO affected its subsequent ability to react with IgG-SpyTag, the ∆N-SpyCatcher-DFO was heated or not for 1 h and then cooled to room temperature and incubated with IgG-SpyTag for 1 h. We stopped the reaction by diluting it 10-fold and storing at −80 °C. Dilution has previously been shown to slow the reaction rate [26]. This was followed by labeling for analysis by a bioanalyzer at 4 °C for 30 min. The bioanalyzer labels free lysines, which should include the active site for the SpyCatcher, therefore quenching the reaction. At a 1:1 molar ratio in the stopped reaction, ~13% of the heavy chain was converted. After 1 min, ~35% of the heavy chain was converted; by 15 min, 94% of the heavy chain was converted; by 30 min, all the heavy chain had been converted (Figure 2C). There was no difference in the reaction of the preheated samples compared to the unheated samples, indicating that the ∆N-SpyCatcher-DFO could be heated prior to labeling with no negative effects (Figure 2C).

### 2.3. ^89^Zr radiolabeling, Characterization, and MicroPET/CT Imaging

**Figure 3:**
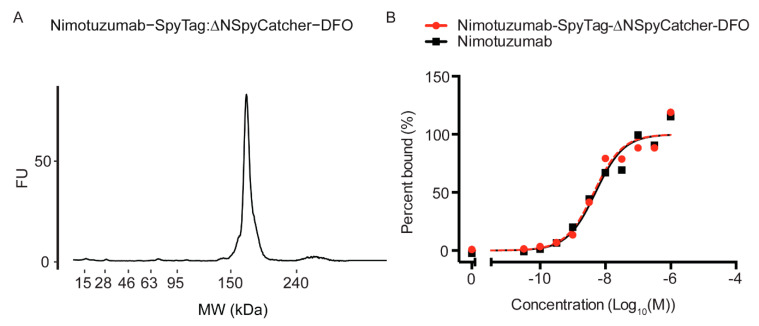
Quality control of the DFO conjugated nimotuzumab-SpyTag-∆N-SpyCatcher. (**A**) Bioanalyzer showing the purity and molecular weight of DFO-nimotuzumab-SpyTag-∆N-SpyCatcher. (**B**) Saturation binding of DFO-nimotuzumab-SpyTag-∆N-SpyCatcher using flow cytometry in epidermal growth factor receptor (EGFR)-positive MDA-MB-468 breast cancer cells.

To radiolabel the IgG with Zirconium-89 (^89^Zr), ligation was performed at a 1:1 ratio of SpyTag:SpyCatcher using either nimotuzumab-SpyTag or control-IgG-SpyTag (10 µM) pre-ligated with ∆N-SpyCatcher-DFO (20 µM). The purity of the nimotuzumab conjugate was 94% pure by bioanalyzer, two ∆N-SpyCatcher-DFO heavier (Figure 3A) and >98% pure on HPLC (Figure S2). Nimotuzumab-SpyTag-∆N-SpyCatcher-DFO (5 ± 1 nM) had the same binding constant as nimotuzumab (5 ± 2 nM) in MDA-MB-468 cells (Figure 3B).

The radiochemical yield of ^89^Zr-nimotuzumab-SpyTag-∆N-SpyCatcher was 76.8 ± 2.1% at a specific activity of 0.5 MBq/µg with a >95% purity (Figure S1). Although not optimized in these studies, this specific activity was similar to that previously reported for ^89^Zr-nimotuzumab using the randomly labeled lysine method [27]. The stability of ^89^Zr-nimotuzumab-SpyTag-∆N-SpyCatcher was determined by instant thin-layer chromatography (iTLC) following incubation at 37 °C in saline and plasma. Starting at 99% radiochemical purity, ^89^Zr-nimotuzumab-SpyTag-∆N-SpyCatcher was >95% after 144 h incubation at 37 °C in saline or mouse plasma (Figure S2).

**Figure 4:**
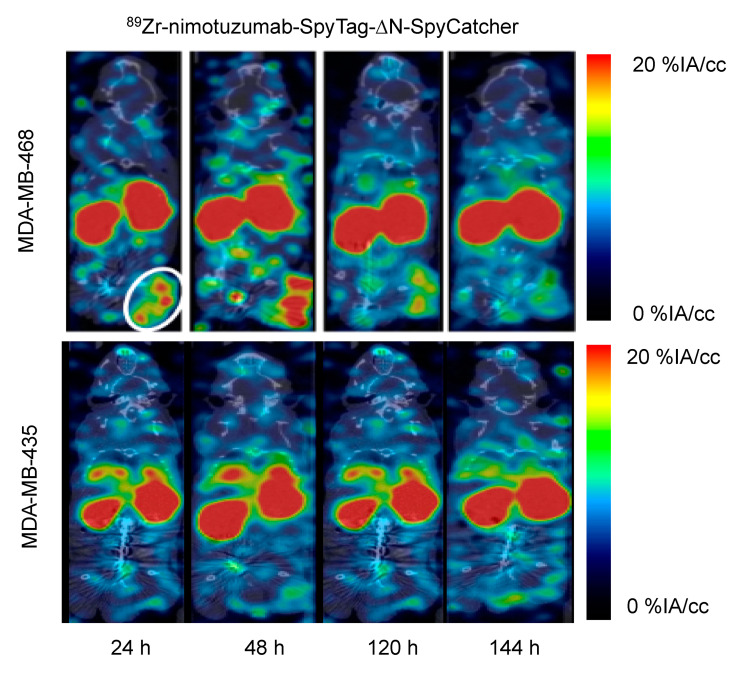
Representative microPET/CT fused images of ^89^Zr-nimotuzumab-SpyTag-ΔN-SpyCatcher tracer injected via tail vein in CD-1 athymic nude mice bearing EGFR-positive (MDA-MB-468) or EGFR-negative (MDA-MB-435) xenografts. Location of xenograft is depicted with a white cycle.

We have previously studied the distribution of full-length nimotuzumab-SpyTag-SpyCatcher in a tumor model using near-infrared imaging (NIR) [28]. The normal tissue distribution of ^89^Zr-nimotuzumab-SpyTag-∆N-SpyCatcher was evaluated in athymic CD-1 nude mice (Table 1). The uptake of ^89^Zr-nimotuzumab-SpyTag-∆N-SpyCatcher was similar with that of the control antibody and showed a high kidney uptake, similar to what was observed in microPET/CT images. The uptake of ^89^Zr-nimotuzumab-SpyTag-∆N-SpyCatcher was also evaluated in mice bearing EGFR-positive (MDA-MB-468) or EGFR-negative (MDA-MB-435) xenografts. MicroPET/CT imaging showed a persistent tumor uptake of ^89^Zr-nimotuzumab-SpyTag-∆N-SpyCatcher in MDA-MB-468 xenografts with a maximum of 6.0 ± 0.6% (IA/cc) at 48 h pi (Figure 4), while the maximum uptake of control antibody ^89^Zr-control-IgG-SpyTag-∆N-SpyCatcher was 2.8 ± 0.8% IA/cc at 48 h pi.

### 2.4. ^225^Ac radiolabeling, Characterization, and In Vitro Cytotoxicity

Nimotuzumab-SpyTag-∆N-SpyCatcher-macropa was prepared and found to be 98% pure in HPLC with <2% aggregates (Figure S3). Radiolabeling resulted in a radiochemical yield of 90.5 ± 1.2% at a specific activity of 0.6 kBq/µg (Figure S3). A radiochemical purity of >95% was obtained following purification. The stability was investigated by iTLC following storage at 37 °C in saline and plasma. ^225^Ac-nimotuzumab-SpyTag-∆N-SpyCatcher tracer starting at 99% radiochemical purity and >95% was observed after 5 days at 37 °C in PBS (Figure S4). In mouse plasma, about 10% of the radiolabel was lost at 5 days post incubation (Figure S4).

Live cell imaging experiments were performed to determine the in vitro anti-proliferative effect of ^225^Ac-nimotuzumab-SpyTag-∆N-SpyCatcher compared with ^225^Ac-control-SpyTag-∆N-SpyCatcher against MDA-MB-468 cells over 48 h. There was a 5-fold difference in the EC_50_ (*p* < 0.01) between nimotuzumab and control, at 3.7 ± 2.6 Bq/mL (0.04 ± 0.03 nM) and 18.5 ± 4.4 Bq/mL (0.2 ± 0.04 nM), respectively.

### 2.5. In Vivo Efficacy of ^225^Ac-Radioimmunoconjugates

**Figure 5:**
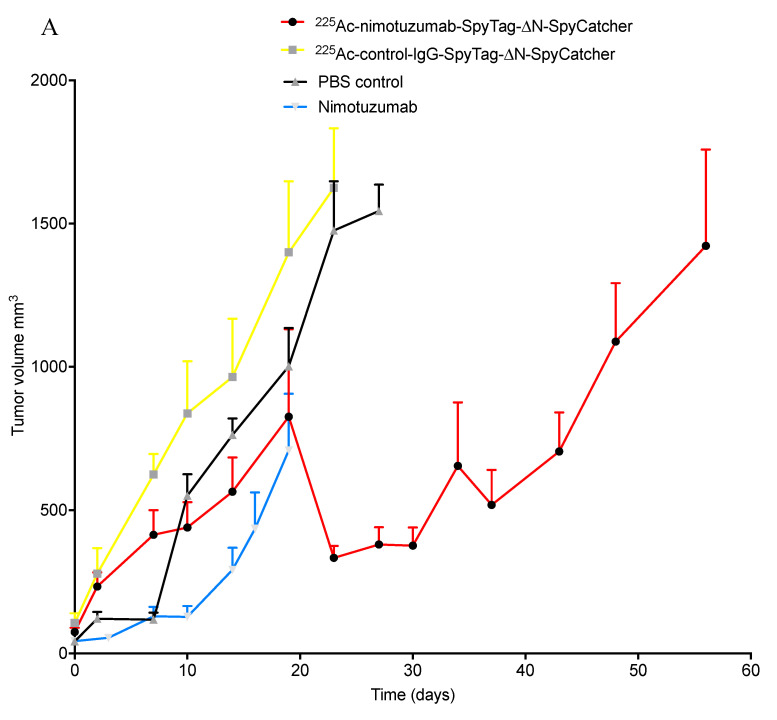

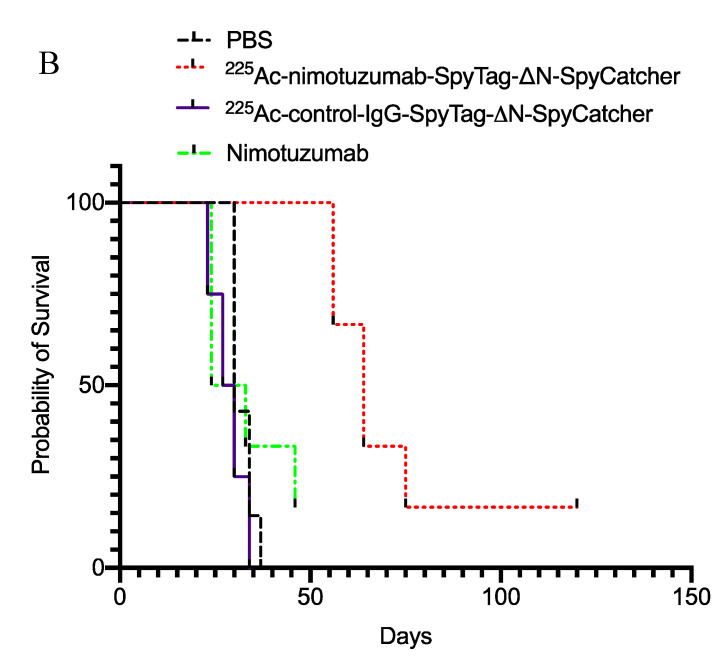
Efficacy of ^225^Ac radioimmunoconjugate. (**A**) Tumor growth curves of treated (^225^Ac-nimotuzumab-SpyTag-ΔN-SpyCatcher) and controls (^225^Ac-control-IgG-SpyTag-ΔN-SpyCatcher; nimotuzumab or PBS). (**B**) Kaplan–Meier survival curves. Study endpoint occurred when the xenograft volume reached 2000 mm^3^. There were no significant differences (*p* > 0.05) in tumor growth and survival between the PBS-treated, nimotuzumab, and ^225^Ac-control-IgG-SpyTag-∆N-SpyCatcher groups, while the ^225^Ac-nimotuzumab-SpyTag-∆N-SpyCatcher was significantly better (*p* < 0.05).

We evaluated the in vivo efficacy of ^225^Ac-nimotuzumab-SpyTag-∆N-SpyCatcher and an ^225^Ac-control in MDA-MB-468 xenograft. Two doses of 450 nCi (16.65 kBq) were administered via a tail vein at day 0 and day 14. This dose was well tolerated, as the mice showed no evidence of weight loss (Figure S4), poor food intake, or morbidity issues. Tumor volume was measured using a digital caliper and mice were sacrificed when the tumor volume exceeded 2000 mm^3^ (Figure 5A). Kaplan–Meier survival curves showed there was no statistical difference between PBS and the ^225^Ac-control antibody treatments (Figure 5B). In the ^225^Ac-nimotuzumab-SpyTag-∆N-SpyCatcher group, 6/8 mice showed response to therapy. From this group, five mice showed tumor relapse after initial response to treatment and had to be sacrificed on day 56 (2 mice), 64 (2 mice), and 75 (1 mouse); one mouse survived to day 120, with a tumor decreasing in volume. Two of the eight mice showed no response to therapy and were sacrificed before day 40. In the ^225^Ac-control-IgG group, all the mice were euthanized by day 29. In the PBS group, all eight mice had reached the study end point (2000 mm^3^) by day 37. The survival (in days) of the ^225^Ac-nimotuzumab-SpyTag-∆N-SpyCatcher group was significantly longer than that of the ^225^Ac-control-IgG and PBS groups (*p* = 0.0017; Log-rank (Mantel–Cox) Test). The median survival of the ^225^Ac-control-IgG-SpyTag-∆N-SpyCatcher, nimotuzumab, and PBS group was 28.5, 28.5, and 30 days, respectively, while that of the ^225^Ac-nimotuzumab-SpyTag-∆N-SpyCatcher group was 64 days (Figure 5B).

## 3. Discussion

This study demonstrates, to our knowledge, the first use of the SpyTag/SpyCatcher for the site-specific labeling of radionuclides to antibodies. We have explored the use of the SpyTag/SpyCatcher system in this context to identify its benefits and limitations. We performed in vitro experiments and determined that the reaction is completed in 15 min at 10 µM of antibody-SpyTag and 20 µM of ∆N-SpyCatcher-DFO. The system is robust in terms of buffer and pH and can withstand high temperatures. These characteristics make it a favorable agent for the reproducible labeling of antibodies with radionuclides. The two radionuclides/chelators ^89^Zr/DFO and ^225^Ac/macropa performed well, indicating the general utility of this method for radiolabeling.

There was no statistical difference in the reactions performed with either nimotuzumab or control IgG, indicating that the identity of the IgG did not affect the reaction and the method should be applicable to any IgG. Additionally, of note is that free ∆N-SpyCatcher-DFO was only observed when it was in molar excess, indicating that there is no need for downstream purification if the IgG-SpyTag is kept in a slight molar excess. The robustness of the ∆N-SpyCatcher to heating and pH, although not exploited in this manuscript, offers unique opportunities to use a hot-conjugation approach, where the radionuclide complex is prepared prior to the bioconjugation step. In that case, the SpyCatcher-chelator would be radiolabeled under harsh conditions and appended to the IgG-SpyTag under gentle conditions (incubating the IgG-SpyTag in PBS with the radiolabeled SpyCatcher for 15 min). Many chelators favor heat; one example of this is ^89^Zr-DOTA, which has an improved stability over many other chelators including ^89^Zr-DFO. However, labeling DOTA with ^89^Zr requires heating at 95 °C for 1 h, a condition incompatible with IgGs [29]. DOTA also forms a stable complex with ^225^Ac, but obtaining high radiochemical yields requires heating the DOTA-conjugated antibody/peptide [30,31,32,33], which was the initial motivation for developing macropa-H2 [34]. The stability of the SpyCatcher compared to the probe being labeled opens up new radionuclides and new labeling strategies not amendable to IgGs, including direct radiolabeling approaches, which are too harsh for IgGs [24]. The method can be extended to prepare conjugates with many repeats of the same compound—for example, to improve the specific activity or the amount of drug delivered [17]. Alternatively, the approach can be used to prepare multimodal theranostic products without interfering with the immunoreactivity of the antibody.

A caveat of using the system to label an IgG-SpyTag is that the SpyCatcher needs to be at 20 µM for efficient labeling. In future studies, to pre-label the SpyCatcher conjugate with the radionuclide we are interested in using the second generation, which requires several-fold less (100 nM) than the one used in this study [26]. This would be in line with the conditions normally used for radiolabeling. An advantage of this strategy is that small amounts of antibody can be efficiently radiolabeled using a pre-labeling strategy. The SpyCatcher-radionuclide can be prepared anywhere and shipped, and the required amount can be reacted on site with an IgG of interest.

As an improvement to our previous work on the site-specific labeling of antibodies with the NIR dye IRDye800CW [28], we chose to use the ∆N-SpyCatcher, which has a reduced immunogenicity [25]. In previous work, we used a 1.5-fold excess, reacted for 3 h, and used additional purification steps to remove unreacted SpyCatcher. Here, we showed that using a 1:1 ratio in labeling can be performed in as little as 15 min and requires no downstream purification. Consistent with our previous work with fluorescent antibodies, we observed a higher than expected uptake in the kidneys. We expected there may be some free SpyCatcher-IRDye800CW, which was one of the reasons we performed the SEC purification of the ∆N-SpyCatcher to completely remove any impurities, yet we still saw an increase in kidney uptake (Figure 4). Given that fluorescence and DFO-conjugated antibodies are different, we now expect that this may be an issue with the maleimide linker, which has previously been shown to be unstable in vivo [35,36]. This apparent instability affected the tumor uptake of ^89^Zr-labeled antibody when compared with the randomly labeled probe ^89^Zr-nimotuzumab [27]. A significantly (*p* < 0.05) lower %IA/cc was observed for the ^89^Zr-nimotuzumab-SpyTag-ΔN-SpyCatcher (6% at 48 h) than for the ^89^Zr-nimotuzumab probe (~12% between 24 and 72 h) for randomly labeled nimotuzumab, as previously reported [27]. Similarly, other major organs such as the blood, kidneys, and liver showed striking differences in uptake compared with the randomly labeled nimotuzumab probe. Future work will explore more stable NHS/NCS chemistries.

In spite of the above in vivo limitations, site-specifically labeled antibody ^225^Ac-nimotuzumab-SpyTag-∆N-SpyCatcher was more effective at controlling tumor growth when compared with control IgG. The rather low therapeutic efficacy of the ^225^Ac-nimotuzumab-SpyTag-∆N-SpyCatcher is likely due to the low in vivo stability of the maleimide bond, which reduced the bioavailability of the construct and hence the tumor uptake. The tumor uptake of ^89^Zr-nimotuzumab-SpyTag-∆N-SpyCatcher in this study was lower than ^89^Zr-nimotuzumab shown previously by our group [27]. The therapeutic effect could be enhanced by optimizing dosing, specifically by adding a third or fourth dose to the treatment regimen and decreasing the time between doses. The doses were administered 14 days apart, and 6/8 mice in the treatment group showed a delay in tumor growth observed for about 30 days, or 14 days following the last dose.

## 4. Experimental Section

### 4.1. General

The chemicals used in the conjugation, radiolabeling, and purification steps were American Chemical Society reagent grade or better. Water and buffers were rendered metal-free by passing them through a column of Chelex-100 resin, 200–400 mesh (Bio-Rad Laboratories, Inc., Hercules, CA, USA), and sterile-filtered through a 0.22 µm filter. The bifunctional chelating agent deferoxamine-maleimide (DFO-maleimide) was obtained from Macrocyclics (Plano, TX, USA). We synthesized macropa-SCN from macropa-NH_2_ obtained from Cornell University (J. J. Wilson Lab, Ithaca, NY, USA) according to a reported procedure [34] under a material transfer agreement. Macropa-SCN was then converted to macropa-PEG_6_-maleimide using a bifunctional NHS-PEG_6_-maleimide, as reported earlier [15]. ^89^Zr-oxalate was received from Washington University School of Medicine (St. Louise, MO, USA). ^225^Ac was supplied as the ^225^Ac-nitrate by the Canadian Nuclear Laboratories (CNL, Chalk River, ON, Canada) under the terms of a research collaboration.

### 4.2. Plasmids

His_6_-Cys-ΔN-SC (Figure 1C) was purchased as a gblock from Integrated DNA Technologies. The plasmid pDEST14-SpyCatcher [23] was purchased from Addgene and digested with *Nde*1 and *Sac*1 to get rid of entire His_6_-SpyCatcher. The PCR-amplified gBlock of His_6_-Cys-ΔN-SC was inserted into the *Nde*1 and *Sac*1 digested pDEST14 backbone using Gibson assembly to generate pDEST14-Cys-ΔN-SC. Previously reported plasmids for the expression of nimotuzumab-SpyTag (pFUSEss-CHIg-Nimotuzumab-hG1-SpyTag/pFUSEss-CLIg-Nimotuzumab-hG1) or control-IgG-SpyTag (pFUSEss-CHIg-Anti-MBP-hG1-SpyTag/pFUSEss-CLIg-Anti-MBP-hG1) [28].

### 4.3. Expression and Purification of His_6_-Cysteine-ΔN-SpyCatcher and Antibodies

His_6_-Cys-ΔN-SpyCatcher expression plasmid was transformed into Rosetta^TM^ (DE3) competent *E. coli* cells (Novagen, Madison, WI, USA) and plated on LB agar containing carbenicillin (100 µg/mL) and chloramphenicol (34 µg/mL). A single colony was picked and cultured overnight in LB with carbenicillin (100 µg/mL) and chloramphenicol (34 µg/mL) at 37 °C. The overnight culture was diluted 100-fold and grown at 30 °C until the OD_600_ reached 0.8, then induced with 0.25 mM IPTG and grown at 30 °C for 4 h. Sample was lysed and purified on a GE Healthcare AKTA FPLC system using a Nickel affinity column (GE healthcare). Nimotuzumab-SpyTag and control-IgG-SpyTag were expressed using the Gibco™ Expi293™ Expression System (Life Technologies, Carlsbad, CA, USA, Catalog Number: A14635) and purified as described previously [28].

### 4.4. Conjugation of Chelator to Cys-∆N-SpyCatcher

Cys-∆N-SpyCatcher (Scheme 1A,B) was first reduced with a 50-fold molar excess of tris(2-carboxyethyl)phosphine (TCEP; Sigma, St. Louis, MO, USA, # C4706) for 2 h at room temperature to form SH-∆N-SpyCatcher (Scheme 1A,B). To this mixture, a 15-fold molar excess of DFO-maleimide or macropa-PEG_6_-maleimide was added and allowed to react overnight at 4 ºC. Excess chelator and TCEP were removed by passing the solution through a 5 mL zeba spin desalting columns (7K MWCO; Fisher Scientific, Waltham, MA, USA). ∆N-SpyCatcher-DFO or ∆N-SpyCatcher-macropa was buffer exchanged to PBS and concentrated with Amicon Ultra centrifugal filters (3 kDa MWCO; Millipore, Burlington, MA, USA). The chelator-labeled SpyCatcher was stored at −80 °C until further use.

### 4.5. Bioconjugation of IgG-SpyTag with Chelator-∆N-SpyCatcher

Unless otherwise indicated, a 1:1 molar ratio of SpyTag to SpyCatcher was used. Nimotuzumab-SpyTag and control IgG-SpyTag (10 µM) were ligated to pre-labeled ∆N-SpyCatcher-DFO or ∆N-SpyCatcher-macropa (20 µM) for 1 h at room temperature in PBS pH 7.2 and terminally sterilized by passing through a 0.22 µm filter (Ultrafree MC; Millipore, Burlington, MA, USA).

### 4.6. Quality Control of Immunoconjugates

The purity of the constructs was determined by HPLC analysis or a bioanalyzer. HPLC was performed using an XBridge Protein size exclusion column (200 Aº 3.5 µM; Waters, Milford, MA, USA) and eluted with 1 × PBS buffer, pH 7.0 at a flow rate of 0.6 mL/min. Proteins were monitored at 280 nm. Molecular weight and purity were characterized by electronic electrophoresis (2100 Bioanalyzer system, Agilent, Santa Clara, CA, USA). The molecular weight (MW) and purity were measured using a high-sensitivity protein 250 Kit (Agilent, Santa Clara, CA, USA, cat # 5067-1575), according to the manufacturer’s protocol. The molecular weight and peak areas were calculated using the 2100 Expert software (Agilent, Santa Clara, CA, USA).

### 4.7. Cell Culture

The human cancer cell line MDA-MB-468 that expresses EGFR and the negative control cell line (no EGFR expression) MDA-MB-435 were obtained from ATCC (Rockville, MD, USA). Cells were propagated by serial passage in 90% DMEM and 90% RPMI medium, supplemented with 10% fetal bovine serum (FBS, Sigma-Aldrich, St. Louis, MO, USA). Cells were grown in a humidified atmosphere with 5% CO_2_ at 37 °C. Prior to in vitro and in vivo use, the cells were detached using trypsin-EDTA.

### 4.8. Flow Cytometry

In vitro binding studies were carried out in EGFR-positive cancer cells MDA-MB-468. 1 × 10^5^ cells were collected and washed with 1 × PBS + 2% FBS. Antibodies were titrated at a minimum of a 10-fold molar excess onto the cells at concentrations between 2000–0.03 nM in a 12-point curve, incubated for 30 min at room temperature followed by 15 min on ice. Cells were washed and suspended in a 1:50 dilution of FITC-labeled Goat F(ab’)2 fragment anti-human IgG (H + L) antibody (Beckman Coulter, Brea, CA, USA) and incubated for 30 min on ice in the dark. Cells were washed and suspended in 1 × PBS + 2% FBS and analyzed using a Gallios flow cytometer (Beckman Coulter, Brea, CA, USA) on the FL1 channel. FlowJo V.10.0.8 was used for analysis and to determine the mean fluorescence intensity. For the fitting and normalization of the mean fluorescence intensity, the top was globally fit and the bottom was fit to the average and then normalized. The K_D_ was calculated based on a non-linear regression curve using GraphPad Prism 6.

### 4.9. Radiolabeling with ^89^Zr and ^225^Ac

Radiolabeling with ^89^Zr was performed as described previously [27]. The purity of the radiolabeled immunoconjugates was determined using size exclusion radio-HPLC and iTLC. Radioactivity was detected using a flow-through radio-HPLC-detector (Flow-RAM, Broomhill, UK). The final solution was formulated in PBS. A radiochemical purity (RCP) of more than 95% was considered sufficient for in vitro and in vivo experiments.

In a typical 1-step procedure, ^225^Ac-nitrate (1.0 MBq) dissolved in 0.05 M of HCl (Optima grade, Fisher scientific, Waltham, MA, USA) was added to a 1.5 mL microtube, and the activity was determined using a dose calibrator. To this, 150 mM of ammonium acetate (pH 6.0, 100 µL) and nimotuzumab-SpyTag-∆N-SpyCatcher-macropa or macropa-control-IgG (833 µg) were added. The pH of the reaction was determined by spotting 1 µL of the reaction mixture onto Hydrion pH paper (range, 5.0–9.0) (Sigma-Aldrich, St. Louis, MO, USA); the pH of a typical reaction was 5.8. The reaction mixture was incubated at 37 ºC on a shaker at 650 RPM for 50 min [37]. After this, a small aliquot (0.5 µL) was spotted on a strip of instant thin-layer chromatography silica gel impregnated paper (iTLC-SG, Agilent Technologies, Santa Clara, CA, USA) to determine the extent of the incorporation of actinium onto the protein using a mobile phase of 50 mM of sodium citrate (pH 5.2). The purification of ^225^Ac-labeled tracer was conducted using Amicon Ultra-4 centrifugal filters (10 K, EMD Millipore, Burlington, MA, USA) with PBS.

### 4.10. Stability of Radioimmunoconjugates

The stability of the radioimmunoconjugates was evaluated in vitro in saline and human plasma. A total of 50 µL of radiolabeled compound was added to 1 mL of human plasma or saline to make a final concentration of 20 MBq/mL (^89^Zr-radiolabeled conjugate) or 1 MBq/mL (^225^Ac-radiolabeled conjugate), followed by incubation at 37 °C for 7 to 10 days (n = 3). Aliquots were taken at different time points and analyzed for radiochemical purity using iTLC [38].

### 4.11. Biodistribution, Tumor Xenografts and MicroPET/CT Imaging

All the animal studies were approved by the University of Saskatchewan Animal Care and Use Committee in accordance with the guidelines set forth on Use of Laboratory Animals (protocol # 20170084). Female athymic CD-1 nude mice were purchased from the Charles River Laboratory (Sherbrooke, QC). The mice were housed under standard conditions in approved facilities with 12 h light/dark cycles and given food and water ad libitum throughout the duration of the studies. For inoculation, MDA-MB-468 or MDA-MB-435 cells were suspended at 5 × 10^7^ cells/mL in a 1:1 mixture of media without FBS: Matrigel (BD Biosciences, Mississauga, ON, Cananda). Each mouse was injected in the right flank with 0.1 mL of the cell suspension.

The biodistribution of ^89^Zr-nimotuzumab-SpyTag-∆N-SpyCatcher and ^89^Zr-control-IgG-SpyTag-∆N-SpyCatcher. was performed in normal female athymic CD-1 nude mice following a tail vein injection of 8–12 MBq (specific activity, 0.5 MBq/µg) of the probes. Mice were sacrificed at 24 and 144 h post injection, and the activity in organs was measured using a gamma counter (Wallac Wizard 1480, PerkinElmer, Waltham, MA, USA) and expressed as the % injected activity per gram (%IA/g).

Mice (n = 4/group) were used for imaging studies when the tumors reached approximately 200–400 mm^3^. Mice bearing MDA-MB-468 or MDA-MB-435 xenografts received an intravenous injection of 8–12 MBq (specific activity, 0.5 MBq/µg) of ^89^Zr-nimotuzumab-SpyTag-∆N-SpyCatcher. At 24, 48, 120, and 144 h post injection, PET and CT images were acquired using a Vector^4^CT scanner (MILabs, Utrecht). Mice were anesthetized using a mixture of isoflurane/oxygen (5% of isoflurane in oxygen), and in vivo whole-body PET/CT images were obtained while anesthesia was maintained (2% of isoflurane). Body temperature, heart rate, and breathing frequency were monitored continuously and kept at normal physiologic values.

The PET scans were acquired in a list-mode data format with a high-energy ultra-high resolution (HE-UHR-1.0 mm) mouse/rat pinhole collimator. Corresponding CT scans were acquired with a tube setting of 50 kV and 480 μA. PET image reconstruction was carried out with a pixel-based order-subset expectation maximization (POS-EM) algorithm that included resolution recovery and compensation for distance-dependent pinhole sensitivity, and was registered on CT and quantified using the PMOD 3.8 software (PMOD, Switzerland).

### 4.12. In Vitro Cytotoxicity

The EC_50_ value of ^225^Ac-nimotuzumab-SpyTag-∆N-SpyCatcher or ^225^Ac-control-SpyTag-∆N-SpyCatcher immunoconjugates was determined using an IncuCyte S3 Live cell imager (Essen BioScience, Ann Arbor, MI). Briefly, 3000–5000 (MDA-MB-468) cells were seeded 24 h prior to treatment in 96-well plates. The next day, the media was removed and washed with PBS. Cells were incubated with IncuCyte^®^ Cytotox Red reagent diluted in complete media (1×, Essen Bioscience, Ann Arbor, MI, USA, Cat #4632) for 3 h before treatment. The cells were treated with different concentrations (3.7–0.028 kBq) of ^225^Ac-nimotuzumab-SpyTag-∆N-SpyCatcher or ^225^Ac-control-IgG-SpyTag-∆N-SpyCatcher and the plate was incubated at 37 °C for 30 min prior to imaging. Live cell images were captured every 2 h using a 10 × objective lens using phase contrast and fluorescence channel. During each scanning, five images were acquired until the end of the experiment (48 h). All the cell images were processed and analyzed using the IncuCyte S3 software. The red fluorescent values were generated and the EC_50_ values for individual compounds were calculated using GraphPad prism 6. The EC_50_ concentration were calculated with reference to a control sample, which represents the concentration that results in a 50% decrease in the cell number/growth/proliferation after 48 h incubation in the presence of an antibody construct [15].

### 4.13. ^225^Ac Radioimmunotherapy

When xenografts averaged 50–100 mm^3^ in volume, the mice were randomized into 4 groups (n = 8 per group). Each group received PBS or two doses of nimotuzumab, ^225^Ac-nimotuzumab-SpyTag-∆N-SpyCatcher, or ^225^Ac-control-IgG-SpyTag-∆N-SpyCatcher via a tail vein on day 0 and 14. The tumor growth was monitored by measuring the greatest length and greatest width of each tumor using digital caliper. Then, the tumor volume was calculated using the formula tumor volume = length × width^2^/2. The study was terminated when the xenograft reached a volume ≥2000 mm^3^, and this was used to determine survival in the different groups using Kaplan–Meier curves. The individual body weights of mice were recorded during the quarantine and experimental period (every other day).

### 4.14. Statistical Analysis

Unless otherwise stated, all data were expressed as the mean ± SD or SEM of at least 3 independent experiments. Statistical comparisons between the experimental groups were performed either via Student *t* tests with Welch correction (2-group comparison) or a 1-way ANOVA with Bonferoni multiple comparison post hoc test (multiple-group comparison). Graphs were prepared and *p* values calculated by using GraphPad Prism (version 6; GraphPad, La Jolla, CA, USA). *p* values of less than 0.5 were considered significant.

## 5. Conclusions

We report for the first time the use of SpyTag/SpyCatcher for the site-specific labeling of radionuclides to antibodies. Nimotuzumab-SpyTag-∆N-SpyCatcher resulted in high radiochemical yields (for ^89^Zr and ^225^Ac) with the excellent preservation of the binding to EGFR. The tracer specifically accumulated in EGFR-positive MDA-MB-468 xenografts, and immunoPET imaging was used to visualize the EGFR expression in vivo. This work represents an exciting option for the site-specific radiolabeling of antibodies for a wide variety of applications. The construct seems unexpectedly unstable in vivo, seemingly due to the unstable maleimide bond. Future studies will investigate more stable conjugation chemistries and the effectiveness of the resulting radiolabeled antibodies in eradicating tumors in vivo.

## Figures and Tables

**Table 1:** Biodistribution of ^89^Zr-nimotuzumab-SpyTag-∆N-SpyCatcher and ^89^Zr-control-IgG-SpyTag-∆N-SpyCatcher in female athymic CD-1 nude mice (*n* ≥ 3 per group), expressed as %IA/g ± SD.

Organs	^89^Zr-Nimotuzumab-SpyTag-∆N-SpyCatcher		^89^Zr-Control-IgG-SpyTag-∆N-SpyCatcher	
	24 h	144 h	24 h	144 h
Kidney	19.2 ± 5.4	24.4 ± 1.8	14.4 ± 2.9	12.6 ± 1.4
Liver	3.8 ± 1.3	2.4 ± 0.3	4.6 ± 1.8	2.0 ± 0.4
Pancreas	0.4 ± 0.1	0.2 ± 0.0	0.2 ± 0.1	0.2 ± 0.0
Spleen	2.2 ± 1.0	2.1 ± 0.1	1.2 ± 0.7	3.5 ± 0.6
Lungs	1.7 ± 0.3	0.7 ± 0.3	0.5 ± 0.3	0.7 ± 0.3
Heart	1.8 ± 1.9	0.4 ± 0.1	0.5 ± 0.2	0.4 ± 0.2
Stomach	0.3 ± 0.2	0.1 ± 0.1	0.2 ± 0.0	0.1 ± 0.0
Intestine	0.4 ± 0.1	0.2 ± 0.1	0.3 ± 0.1	0.2 ± 0.0
Blood	3.6 ± 0.8	1.0 ± 0.3	1.1 ± 0.3	1.0 ± 0.8
Bone	0.7 ± 0.2	0.3 ± 0.0	0.4 ± 0.2	0.4 ± 0.0
Muscle	0.3 ± 0.0	0.2 ± 0.0	0.2 ± 0.1	0.2 ± 0.2
Skin	1.2 ± 0.2	0.7 ± 0.1	0.8 ± 0.2	0.8 ± 0.2

## Supplementary Materials

The following are available online at https://www.mdpi.com/2072-6694/12/11/3449/s1: Figure S1: Representative size exclusion (SEC) HPLC profile of 89Zr-nimotuzumab-SpyTag-ΔN-SpyCatcher. UV channels at 220 and 280 nm show “cold” antibody and radiometric channel shows the radiolabeled antibody with a purity > 95%, Figure S2: Stability of 89Zr-nimotuzumab-SpyTag-∆N-SpyCatcher in saline and human plasma at 37 °C for different time points, Figure S3: Representative size exclusion (SEC) HPLC profile of 225Ac-nimotuzumab-SpyTag-∆N-SpyCatcher. UV channels at 220 and 280 nm show “cold” antibody and radiometric channel shows the radiolabeled antibody with a purity > 95%, Figure S4: Stability of 225Ac-nimotuzumab-SpyTag-∆N-SpyCatcher in saline and human plasma at 37 °C at different times post incubation. Average body weight of mice during treatment for the different groups.

## Abbreviations

HEPES	4-(2-hydroxyethyl)-1-piperazineethanesulfonic acid
CT	Computed tomography
DFO	Desferrioxamine
EC_50_	Effective concentration
EGFR	Epidermal growth factor receptor I
EDTA	Ethylenediaminetetraacetic acid
LET	linear energy transfer
PET	Positron emission tomography
