# Supplementary material for: Nimotuzumab Site-Specifically Labeled with 89Zr and 225Ac Using SpyTag/SpyCatcher for PET Imaging and Alpha Particle Radioimmunotherapy of Epidermal Growth Factor Receptor Positive Cancers
Source: Cancers (Basel). 2020 Nov 20;12(11):3449. doi: 10.3390/cancers12113449 (PMC7699480; doi:10.3390/cancers12113449)
Supplement: Supplementary file 1 [file cancers-12-03449-s001.pdf]

cancers

MDPI

Supplementary Materials: Nimotuzumab Site-Specifically Labeled with ^{89}Zr and ^{225}Ac Using SpyTag/SpyCatcher for PET Imaging and Alpha Particle Radioimmunotherapy of Epidermal Growth Factor Receptor Positive Cancers

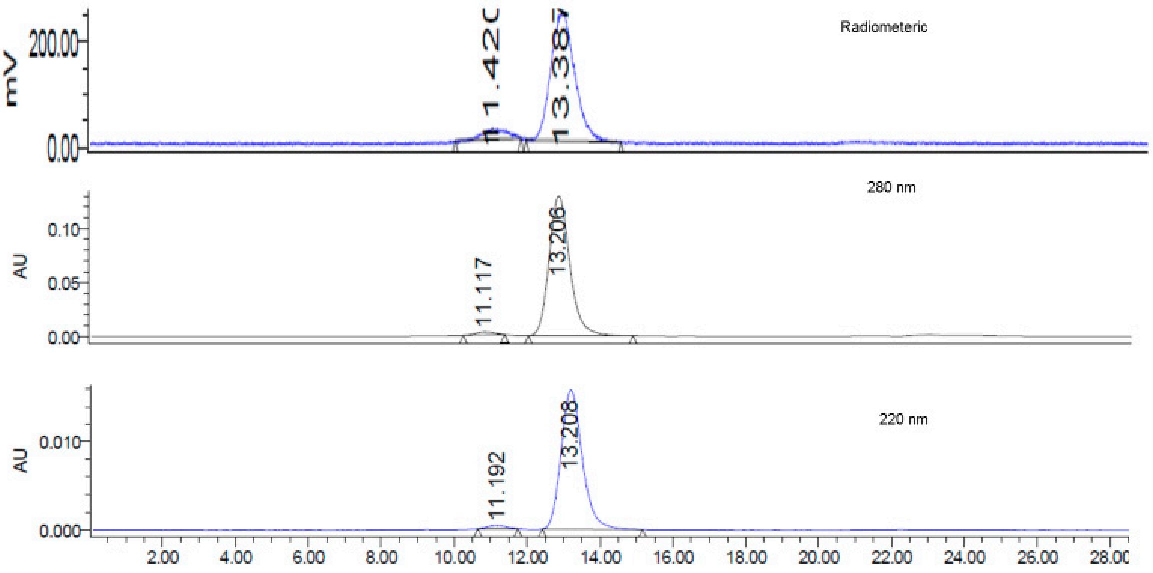

Figure S1. Representative size exclusion (SEC) HPLC profile of ^{89}Zr-nimotuzumab-SpyTag-ΔN-SpyCatcher. UV channels at 220 and 280 nm show "cold" antibody and radiometric channel shows the radiolabeled antibody with a purity > 95%.

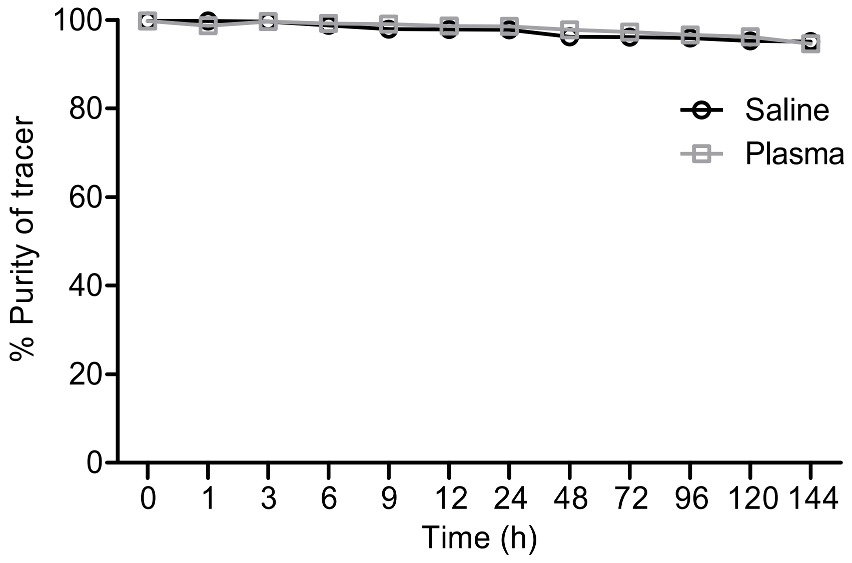

Figure S2. Stability of ^{89}Zr-nimotuzumab-SpyTag-ΔN-SpyCatcher in saline and human plasma at 37 °C for different time points.

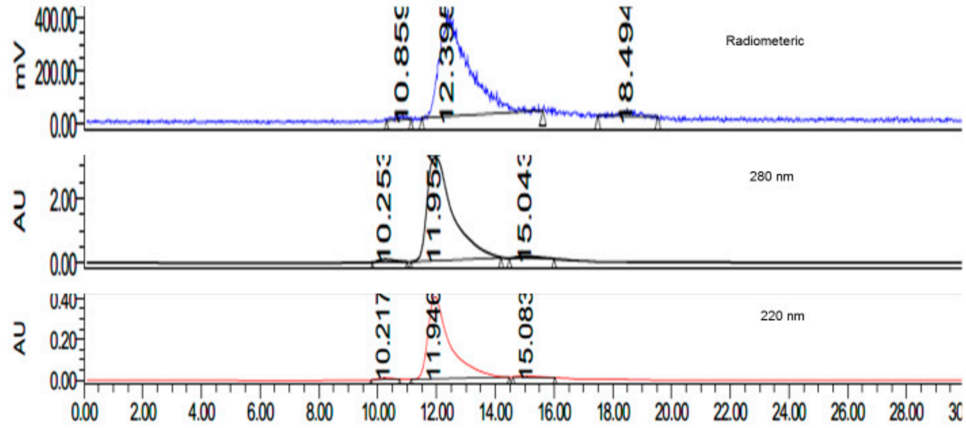

Figure S3. Representative size exclusion (SEC) HPLC profile of ^{225}Ac-nimotuzumab-SpyTag-ΔN-SpyCatcher. UV channels at 220 and 280 nm show "cold" antibody and radiometric channel shows the radiolabeled antibody with a purity > 95%.

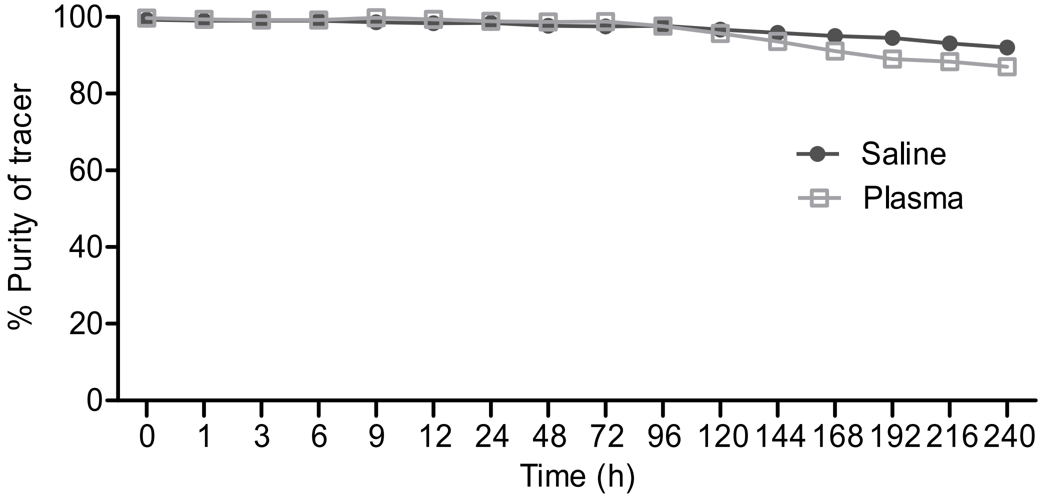

Figure S4. Stability of ^{225}Ac-nimotuzumab-SpyTag-ΔN-SpyCatcher in saline and human plasma at 37 °C at different times post incubation.

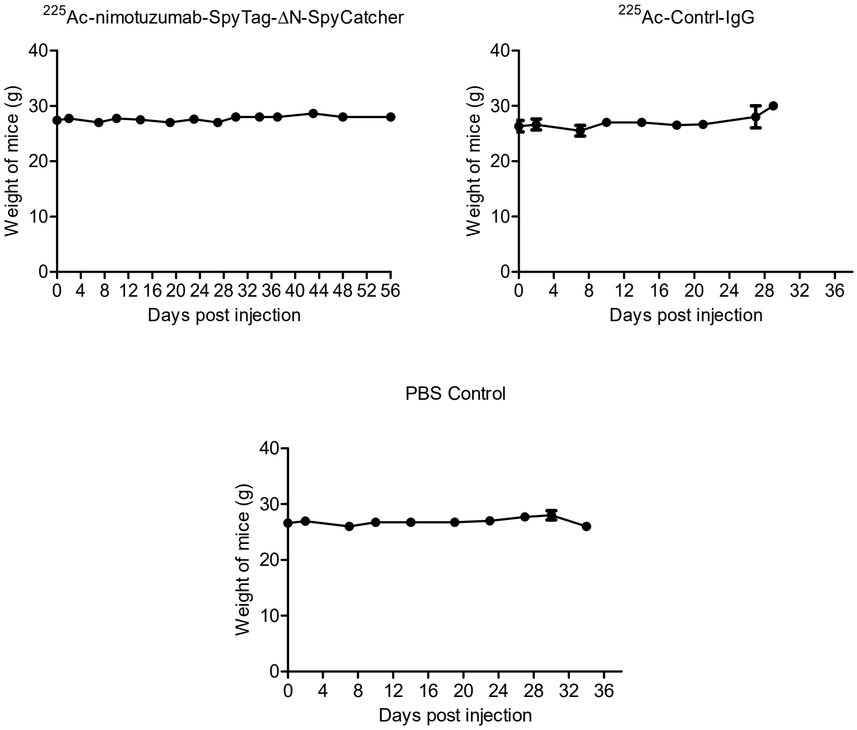

Figure S5. Average body weight of mice during treatment for the different groups.

© 2020 by the authors. Licensee MDPI, Basel, Switzerland. This article is an open access article distributed under the terms and conditions of the Creative Commons Attribution (CC BY) license (http://creativecommons.org/licenses/by/4.0/).
